# High-affinity nitrogen-doped graphene quantum dots for selective *in vivo* and *ex vivo* detection of amyloid-β plaques in an Alzheimer's disease rat model

**DOI:** 10.1039/d5ra07458d

**Published:** 2026-02-19

**Authors:** Mina Bahman, Mohammad-Reza Milani-Hosseini, Seyed Behnamedin Jameie

**Affiliations:** a Research Laboratory of Real Samples Analysis, Faculty of Chemistry, Iran University of Science and Technology Tehran Iran smrezamilani@gamil.com jameie.sb@iums.ac.ir; b Neuroscience Research Center, Iran University of Medical Sciences Tehran Iran behjame@gmail.com; c Department of Anatomical Sciences, Faculty of Medicine, Iran University of Medical Sciences Tehran Iran

## Abstract

Early and precise detection of the amyloid-β (Aβ) aggregates is a critical factor in understanding Alzheimer's disease (AD) pathology. Current fluorescent probes for detecting Aβ plaques suffer from poor photostability, low selectivity, and ineffective blood–brain barrier (BBB) permeability, hindering *in vivo* imaging efficacy. Herein, we develop the first demonstration of nitrogen-doped graphene quantum dots (N-GQDs) as multifunctional fluorescent probes enabling sensitive, selective, and real-time *in vivo* and *ex vivo* imaging of Aβ aggregates in an AD rat model that overcomes previous limitations. Unlike conventional organic dyes, our N-GQDs combine high photostability with enhanced biocompatibility (cytotoxicity <10% at 250 µg mL^−1^) and high quantum yield (57.3%). Their nanoscale size (7.4 nm) facilitates efficient BBB penetration and rapid clearance, addressing a major challenge in existing Aβ imaging agents. Nitrogen doping increases the affinity and selective binding interactions of GQDs with Aβ aggregates by introducing active sites and modifying their electronic structure. N-GQDs showed a fluorescence enhancement specifically upon binding to Aβ_25–35_ aggregates, providing sensitive detection at concentrations as low as 1.6 µM. Molecular docking analysis confirmed a strong and stable interaction (−57.4 kcal mol^−1^) between N-GQDs and Aβ_25–35_ aggregates, supporting the observed selectivity. Following intravenous injection of the N-GQDs, the fluorescent intensity in the brain of the AD model rats showed a ∼2-fold increase compared to that of control rats, consistent with *ex vivo* biodistribution studies. These findings establish N-GQDs as the first graphene-based platform for non-invasive, selective *in vivo* detection of Aβ plaques, offering them as a diagnostic agent for AD pathology.

## Abstract 2

Early and precise detection of the amyloid-β (Aβ) aggregates is a critical factor in understanding Alzheimer's disease (AD) pathology.

## Graphical abstract

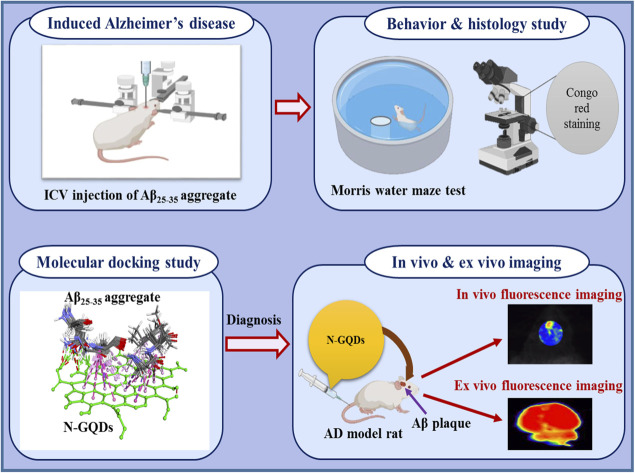

## 1 Introduction

Alzheimer's disease (AD) is the most widespread neurodegenerative disease, distinguished by memory decline, cognitive impairment, and confusion, representing 60–70% of dementia cases, particularly in the older population.^1–3^ Presently, AD can be recognized *in vivo* in patients at the preclinical stage, for instance, by the disease's genetic or biological signature.^4^ The hallmark pathologies of AD include intracellular tau accumulation and extracellular amyloid β-protein (Aβ) aggregation, and these changes begin at least 20 years before symptoms appear.^5,6^ The accumulation of cerebral amyloid plaques happens before the appearance of clinical signs, and the aggregation of Aβ is considered a key factor for AD pathogenesis. Furthermore, Aβ is a significant biomarker for both early diagnosis and monitoring therapeutic response.^7^ Oligomeric forms of Aβ, primarily Aβ_1–40_ or Aβ_1–42_, are commonly found in AD patients. These peptides have been widely used in the development of AD models to understand the physiopathology of the disease because they have been demonstrated to cause inflammation, learning deficits, and long-lasting impaired synaptic plasticity.^8^ Studies suggest that Aβ_25–35_ is produced through enzymatic cleavage of Aβ_1–40_ in AD patients. This particular undecapeptide, Aβ_25–35_, has drawn considerable interest because of its inherent β-sheet structure and its ability to induce memory impairments, neurite degeneration, synaptic dysfunction, and neuronal death, similarly to Aβ_1–40_ and Aβ_1–42_, but with higher solubility and potency.^9,10^ Consequently, intracerebroventricular (ICV) injection of Aβ_25–35_ is most commonly used to create non-transgenic rat models of amyloidosis.

In recent decades, neuroimaging techniques have been introduced for selective and specific imaging and monitoring of Aβ plaque, including magnetic resonance imaging (MRI),^11,12^ radiation-based positron emission tomography (PET),^13,14^ and single-photon emission-computed tomography (SPECT).^15,16^ Despite PET and SPECT being the predominant imaging procedures for neurodegenerative disease, their utility is constrained by cost implications, usage of potentially hazardous radiolabeled substances, necessity for advanced equipment, and implementation of complex data collection and analysis protocols. On the other hand, the MRI method exhibits limited spatial resolution and insufficient sensitivity to recognize morphological differences between disease biomarkers and the adjacent tissues.^17^ Currently, fluorescence diagnostic technology has emerged as an appealing and promising alternative for diagnosing and tracking the progression of neurodegenerative disorders because of its speed, non-invasiveness, sensitivity, simplicity, real-time capability, cost-effectiveness, and high resolution.^18,19^

Different tissue staining techniques, including Congo red (CR) and thioflavin T (ThT), have been commonly used to stain Aβ aggregates and fibrils to diagnose AD. CR and ThT are not supposed to be precise methods for the diagnosis of AD because they are not capable of penetrating the blood–brain barrier (BBB) due to the charge on these molecules, and they are not able to label with the monomeric Aβ.^20–22^ To date, several fluorescence probes for *in vivo* imaging of Aβ aggregates have been reported.^23–28^ However, the low BBB penetration and high lipophilicity of these probes have restricted their application in fluorescence-based AD diagnostics. Therefore, a small, biocompatible, and highly selective fluorescence probe that could rapidly penetrate the BBB is critical for the early detection of Aβ plaques.

Recently, graphene quantum dots (GQDs), a novel class of fluorescent carbon nanomaterial consisting of one or few graphene layers with lateral sizes under 100 nm, have received enormous interest due to their excellent photostability, low toxicity, superb biocompatibility, small size, high water solubility, tunable photoluminescence (PL) properties, good surface bonding employing the π–π conjugated system, as well as several surface functional groups at the edges or basal plane of GQDs.^29–33^ In addition, the previous studies indicate that GQDs could penetrate the BBB *in vivo* and are eliminated from the biological system by renal excretion.^34–37^ These outstanding features make GQDs an attractive material in biomedical fields, especially for *in vitro* and *in vivo* bioimaging,^38,39^ diagnostics,^40,41^ and therapy.^42,43^ Although previous research has demonstrated that GQDs can inhibit Aβ aggregation,^44,45^ their direct application for *in vivo* diagnosis of Aβ plaques remains unexplored.

Heteroatom doping is a valuable approach to further enhance the diagnostic capabilities of GQDs. Nitrogen (N) doping of GQDs can significantly modulate their intrinsic features, such as electronic properties and surface chemistry. Among various heteroatoms, nitrogen is considered a suitable element for heteroatom doping in GQDs because it has a similar atomic size to the carbon atom and five valence electrons that can form stable bonds with the carbon atom.^46,47^ Doping N atoms with GQDs (N-GQDs) can result in increased fluorescent quantum yield, additional active sites, and improved biocompatibility, which ensures their advantages in biological applications.^48^ More importantly, in comparison to un-doped GQDs, N-doping can enhance the interaction between GQDs and Aβ aggregates through hydrogen bonding and electrostatic interactions, thereby improving both the sensitivity and selectivity of Aβ detection.

To our knowledge, the use of N-GQDs has not previously been investigated as *in vivo* fluorescence probes for the selective and sensitive detection of Aβ plaques. Unlike earlier studies mainly focused on conventional dyes such as thioflavin dyes or fluorescent ligands with limited selectivity, poor biocompatibility, and low BBB permeability, our work introduces a new N-GQDs-based Aβ imaging probe that overcomes these limitations. In this study, we present for the first time the application of N-GQDs as highly selective and biocompatible fluorescent probes for both *in vivo* and *ex vivo* detection of Aβ plaques in an AD rat model. We developed water-soluble N-GQDs through a facile, cost-effective pyrolysis method. Fluorescence spectroscopy analyses revealed a strong turn-on response and remarkable specificity of N-GQDs toward Aβ_25–35_ aggregates *in vitro*. Molecular docking studies elucidated the binding mechanism and indicated strong affinity of N-GQDs for Aβ_25–35_ aggregates. Notably, we confirmed their diagnostic potential in an AD rat model, demonstrating real-time, non-invasive detection of Aβ plaques in both *in vivo* and *ex vivo*. This research supposes that N-GQDs serve as a novel class of Alzheimer's imaging probes, offering a unique combination of high selectivity, photostability, quantum yield, BBB penetrability, and biocompatibility that distinguishes them from existing fluorescent probes. These features provide significant advances for early and accurate diagnosis of AD.

## 2 Experimental section

### 2.1. Materials

The materials employed in this research are listed below: citric acid (Sigma-Aldrich, Merck), urea (Sigma-Aldrich, Merck), Aβ_25–35_, Aβ_1–40_, and Aβ_1–42_ (Abcam, UK), ketamine and xylazine (Alfasan, Ntherlands), dimethyl sulfoxide (DMSO, Gibco, USA), Dulbecco's modified eagle's medium (DMEM, Gibco, USA), penicillin-streptomycin (Gibco, USA), fetal bovine serum (FBS, Gibco, USA), trypsin (Gibco, USA), 3-(4,5-dimethylthiazol-2-yl)-2,5-diphenyltetrazolium bromide (MTT, Sigma-Aldrich, Merck), phosphate buffer solution (PBS, pH 7.4, Gibco, USA), paraformaldehyde (PFA, Sigma-Aldrich, Merck), Congo red (Sigma-Aldrich, Merck). All of the chemicals were analytical grade and applied directly without additional purification.

### 2.2. Apparatus

UV-vis absorption spectra were measured on a PG Instruments T80+ UV/vis spectrophotometer utilizing a quartz cuvette with a 1 cm optical path. Photoluminescence (PL) spectra were recorded using a PerkinElmer LS50 luminescence spectrometer. Fourier transform infrared (FT-IR) spectrum was obtained on a Shimadzu-8400S spectrometer in the 400–4000 cm^−1^ range at ambient temperature with KBr pellets. Energy-dispersive X-ray (EDX) spectra were acquired on the Philips XL30 scanning electron microscope (SEM). The morphology of N-GQDs was examined using transmission electron microscopy (TEM, Philips, CM120, 100 kV). The pH of the solutions was determined with a Metrohm digital pH meter. X-ray diffraction (XRD) was performed on a Philips diffractometer (model X'Pert MPD). The Raman spectrum was operated on an Almega Thermo Nicolet Dispersive Raman Spectrometer with a 532 nm laser. The KODAK imaging system (System FX Pro) was used to obtain both *in vivo* and *ex vivo* imaging.

### 2.3. N-GQDs synthesis

Carbonization and pyrolysis processes were used in a single-step to create the N-GQDs. A typical synthesis involved adding 0.6 g of citric acid and 0.3 g of urea into a crucible and stirring well.^49^ After that, the mixture was heated to 200 °C in a blast drying oven for 30 min, and it was then cooled to ambient temperature. A brownish-black solid N-GQD powder was the product of the reaction. The N-GQD powder was obtained and dissolved in ultrapure water to create a clear yellow solution. After being centrifuged several times at 10 000 rpm, the N-GQDs aqueous solution was filtered through a 0.22 µm microporous membrane to remove the large dots. Finally, the prepared N-GQDs were stored at 4 °C for further studies.

### 2.4. Quantum yield determination

The quantum yield (QY) of the N-GQDs was measured using quinine sulfate in 0.1 M sulfuric acid (H_2_SO_4_) as a reference standard. Five different concentrations of both N-GQDs and quinine sulfate solutions were prepared. To reduce the re-absorption effects, the absorbance values of the N-GQDs and quinine sulfate were maintained below 0.1 at the excitation wavelength. Fluorescence emission spectra were obtained for each concentration, and the integrated emission intensity was plotted against the absorbance at the excitation wavelength. The slope of the linear regression for the N-GQDs and the standard was calculated from these plots. Then, the QY of the N-GQDs was measured relative to the reference standard using the slope method according to the eqn (1):

1 *Φ*_X_ = *Φ*_R_ (*m*_X_/*m*_R_) (*η*_X_/*η*_R_)^2^

where *Φ*_R_ indicates the QY of quinine sulfate (54.0%), *m*_X_ and *m*_R_ denote the slopes of the integrated emission intensity *versus* absorbance plots for N-GQDs and quinine sulfate, respectively, while *η*_X_ and *η*_R_ refer to the refractive index of the solvents used for N-GQDs and quinine sulfate.

### 2.5. Formation of Aβ species

Aβ peptides (Aβ_25–35_, Aβ_1–40_, and Aβ_1–42_) were prepared separately as monomeric, oligomeric, and aggregated forms according to established protocols. Monomeric stock solutions were obtained by dissolving each peptide in hexafluoroisopropanol (HFIP).^50^

Aβ_25–35_ oligomers were generated by diluting a 5 mM stock solution of Aβ_25–35_ in DMSO with ice-cold PBS to a concentration of 100 µM. The mixture was vortexed for 30 seconds and then incubated at 4 °C for 72 h.^51^

To obtain Aβ_1–40_ and Aβ_1–42_ oligomers, 1.0 mg of each peptide was dissolved in 400 µL HFIP and incubated at room temperature for 15 min. An aliquot (100 µl) was diluted into 900 µl deionized water in a siliconized tube and incubated for an additional 15 min. The mixture was centrifuged at 14 000 × G for 15 min, and the supernatant was collected. Residual HFIP was removed by gentle nitrogen stream for 10 min, and the solution was then stirred at 500 rpm at 22 °C for 24–48 h to allow oligomer formation.^50^

Aβ_25–35_ aggregates were obtained by dissolving them in sterile double-distilled water at desirable concentrations and then incubating them at 37 °C for 4 days.^52^

To form Aβ_1–40_ and Aβ_1–42_ aggregates, each peptide (1.0 mg) was dissolved in 1.0 mL of 1% ammonium hydroxide. The solution (100 µl) was diluted 1 : 10 in PBS (pH = 7.4) and stirred at room temperature for 72 h.^25^

### 2.6. Fluorescence testing of the N-GQDs with Aβ_25–35_ aggregates

To investigate the *in vitro* interaction between the N-GQDs and Aβ_25–35_ aggregates, 0.1 mg mL^−1^ of the N-GQDs solution (2 mL, pH = 7.4) was mixed with different concentrations of Aβ_25–35_ aggregates (2–20 µM). Then, we incubated the prepared mixture at room temperature for 5 min; it was placed in a quartz cuvette, and the fluorescence measurements at different concentrations were obtained using an excitation wavelength of 360 nm.

### 2.7. Selectivity and interference study

The selectivity of N-GQDs toward Aβ_25–35_ was investigated by fluorescence spectroscopy and compared with Aβ_1–40_ and Aβ_1–42_ in their monomeric, oligomeric, and aggregated forms. Selectivity experiments were performed both in the absence and presence of potential interfering Aβ species under identical experimental conditions. Each Aβ species was used at a concentration of 10 µM, while the concentration of N-GQDs was fixed at 0.1 mg mL^−1^. Fluorescence measurements were recorded at an excitation wavelength of 360 nm after incubation of N-GQDs with each Aβ species for 5 min at room temperature. Interference studies were conducted by measuring the fluorescence response of N-GQDs toward Aβ_25–35_ in the presence of competing Aβ species at the same concentration (10 µM each).

### 2.8. Molecular modeling

The docking simulations of Aβ_25–35_ and N-GQDs were conducted by the AutoDock Vina software, which is a popular tool for molecular docking and is well known for its precision and speed. The protein data bank provided the protein structure required for the docking analysis (PDB ID: 1QCM). Preparation of the protein structure involved adding hydrogens, assigning charges, and removing water molecules and other ligands present in the crystal structure using AutoDock Tools. Similarly, the N-GQDs structure was prepared by adding hydrogens and assigning charges using the same software. The grid box size was set to *X* = 17, *Y* = 15, and *Z* = 15, and the center was set to *X* = 2.38, *Y* = 0.99, and *Z* = −1.81.

### 2.9. Cell culture and measurement of cell viability

The differentiated PC12 cells were acquired from the Pasteur Institute (Tehran, Iran). The PC12 cells were cultured in DMEM containing 10% FBS, penicillin (100 units per mL), and streptomycin (100 µg mL^−1^), and incubated at 37 °C with 5% CO_2_ in a humidified atmosphere. The viability of cells was evaluated by the MTT assay as previously described.^53^ The PC12 cells were plated into 96-well plates at a density of 5 × 10^3^ cells in each well. Following 24 h of cell attachment, cells were exposed to various concentrations of N-GQDs for 24 h. Then, 10 µl of MTT solution (5 mg mL^−1^) was added to each well and incubated at 37 °C for 4 h. After removing the culture medium, 100 µl of DMSO was added to each well to dissolve the formed formazan crystals. The plate was shaken for 10 min, and then the absorbance was recorded with a microplate reader at 570 nm. The viability of cells in each well was presented as a percentage compared with the control group cells. The experiments were repeated three times and carried out in triplicate.

### 2.10. Experimental animal groups

Male Wistar rats with a weight range between 200 and 250 g (*n* = 24) were purchased from the Animal House of Iran University of Medical Science (Tehran, Iran). The animals were maintained under a 12-h light–dark cycle, with lights turned on at 7:00 a.m., in a temperature environment of 21 ± 2 °C. They were housed in groups of four in stainless steel cages with free access to water and food without limitation. The rats were then separated into two experimental groups (*n* = 12 for each group): (1) the control group (without any intervention) and (2) the Aβ_25–35_ model group, which received ICV administration of Aβ_25–35_.

### 2.11. Stereotaxic surgery and ICV injection of Aβ_25–35_

To establish the AD animal model, rats were anesthetized *via* intraperitoneal (i.p.) administration of a ketamine (100 mg kg^−1^) and xylazine (10 mg kg^−1^) mixture. They were then kept on a stereotaxic apparatus (Stoelting Co., Wood Dale, IL, USA). Using the Paxinos and Watson rat brain atlas,^54^ stereotaxic coordinates were set at −0.8 mm anterior–posterior, ±1.5 mm lateral from the bregma, and −4.0 mm dorsoventral. After shaving the hair of the skull, a midline sagittal incision was created in the scalp. Burr holes were carefully drilled in the skull on both sides above the lateral ventricles. An electrically protected heating pad was employed to keep the rats' body temperature at 37 °C during the Aβ_25–35_ injection process. The bilateral ICV injection of aggregated Aβ_25–35_ with a concentration of 2.5 µg µl^−1^ (5 µl per side) was performed by using a Hamilton syringe at a flow rate of 1 µl min^−1^ (Hamilton, Reno, Nevada) with a 26-gauge stainless steel needle. The needle remained in place for five minutes after the injection. After surgeries, animals received penicillin (50 mg kg^−1^) and ketoprofen (0.5 mg kg^−1^) in a single dose and were then kept in individual cages with unlimited access to food and water.

### 2.12. Morris water maze test

The Morris water maze (MWM) test is widely utilized in animal models to evaluate cognitive function, especially spatial learning and memory.^55,56^ Following the protocol established by Morris' study, the MWM test was conducted after 14 days of Aβ_25–35_ injection.^57^ In the MWM, a circular pool (diameter = 110 cm; height = 70 cm) filled with tap water (21 ± 1 °C) was separated equally into four quadrants, which were identified as the East, West, North, and South. In the center of one of these quadrants, a hidden circular platform was placed. In this research, the test was carried out for four consecutive days. The study was divided into two stages: (i) an acquisition phase that involves four trials daily to investigate spatial learning, and (ii) a single probing trial to evaluate memory retention. During the acquisition phase, each trial involved dropping the animal into the water in a different quadrant and giving it 120 seconds to find the hidden platform. The rats stayed on the platform for 30 seconds after discovering the hidden platform, and escape latency was measured at 120 seconds. The hidden platform was removed after 24 hours of the last acquisition trial, and the probe trial was then performed for 120 seconds. To assess spatial learning performance, we measured the time the animals stayed on the platform (escape latency) and the distance they traveled to reach the hidden platform (distance traveled). For the probe test, the parameters examined were the time spent in the target quadrant and the latency of the first entry into it. The swimming routes of animals were automatically tracked using a video tracking system (EthoVision, Noldus, version 14).

### 2.13. Hippocampus Congo red staining

A histological study was performed on hippocampal tissues collected from randomly selected rats in each group (*n* = 4 per group). The animals were deeply anesthetized by i.p. injection of ketamine (100 mg kg^−1^) and (10 mg kg^−1^) xylazine, followed by transcardial perfusion using 0.9% saline solution and subsequently 4% paraformaldehyde (PFA) in phosphate buffer saline (PBS, pH = 7.4) at a gradual and steady rate. The extracted brains were immersed overnight in 4% PFA for fixation and then transferred to a 30% sucrose solution for cryoprotection for 3 days. Using a cryostat (Leica, Nussloch, Germany), brain sections of 20 µm thickness were obtained and mounted onto glass slides. Congo red staining was used to detect Aβ plaques by a modified Highman's Congo red staining protocol.^58^ In the assay, brain sections were initially hydrated in distilled water for 2 min and then stained in 0.5% Congo red solution (prepared in 50% ethanol) for 15–20 min. Afterward, the sections were rinsed in distilled water and differentiated (5–10 quick dips) in an alkaline alcohol solution (1% sodium hydroxide in 50% ethanol). After rinsing in tap water for 2 min, section counterstaining was carried out with Gill's hematoxylin for 30 s. Then, the sections were rinsed again in tap water for 2 min, dehydrated using 95% and 100% ethanol (repeated twice) for 3 min, cleared in xylene, and finally coverslipped. Congo red-stained sections were studied by light microscope.

### 2.14. *In vivo* fluorescence imaging

*In vivo* imaging with fluorescent mode was carried out by a KODAK imaging system (System FX Pro) at the Preclinical Core Facility (TPCF) based at the Tehran University of Medical Sciences. Different excitation and emission wavelengths were tested, and finally, fluorescence images were acquired with optimal conditions of 470 nm for excitation and 535 nm for emission and an exposure time of 5 min. The emitted light from the rats was identified by the KODAK camera system, then integrated, converted into digital form, and visualized.

Before background imaging, AD model rats (*n* = 4) and age-matched control rats (*n* = 4) were shaved and then followed by intravenous injection of freshly produced N-GQDs (5 mg kg^−1^ in PBS). Brain fluorescence signals were obtained before and at 30, 60, 120, 180, 240, and 360 minutes following the intravenous injection of the N-GQDs. A region of interest (ROI) was selected around the brain area to assess the imaging results. Before imaging, the rats were placed in the imaging stage under anesthesia with isoflurane gas supplemented with oxygen gas. The imaging data was investigated by Living Image software. Every rat's background fluorescence intensity was used to normalize the brain fluorescence intensity measured at each time point (*i.e.*, *F*(*t*)/*F*(pre)), where *F*(*t*) represents the fluorescence signal at the particular time point and *F*(pre) represents the background fluorescence intensity.

### 2.15. *Ex vivo* fluorescence imaging

AD model rats (*n* = 4) and age-matched control rats (*n* = 4) were intravenously injected with N-GQDs (5 mg kg^−1^ in PBS). To evaluate the N-GQDs distribution in the brain and key organs (liver, kidney, and spleen), rats were sacrificed 30 min after the N-GQDs injection, and then fluorescence images of the extracted organs were obtained.

### 2.16. Statistical analysis

To perform statistical analysis, SPSS software (version 27) was employed. A student's *t*-test, one- and two-way analysis of variance (ANOVA), followed by Tukey's multiple tests, was performed to compare between groups. The data have been displayed as mean ± standard deviation (SD), and a statistically significant *P*-value was regarded as below 0.05.

## 3 Results and discussion

### 3.1. The N-GQDs characterization

**Fig. 1:**
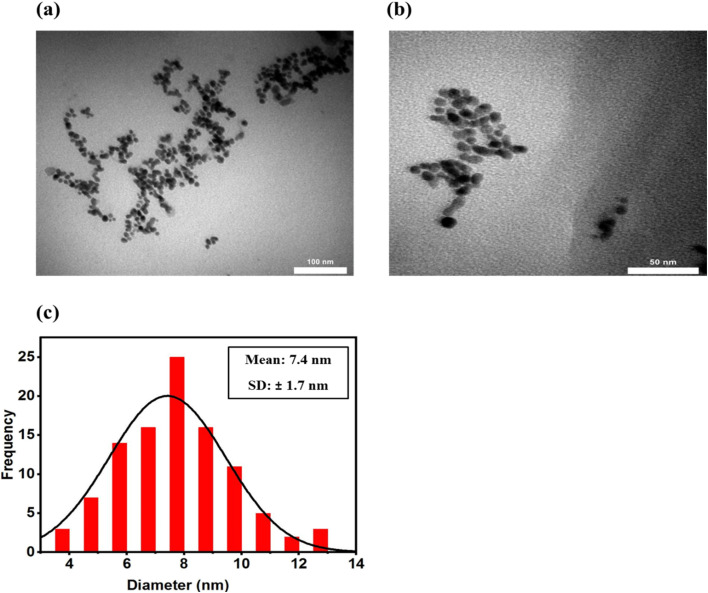
The N-GQDs TEM images with different scale bars: (a) 100 nm (b) 50 nm. (c) The particle size distribution histogram of the N-GQDs.

The N-GQDs were prepared using the pyrolysis process mentioned in the previous part (2.3). Different characterization techniques were used to identify their structural and morphological features. TEM analysis was utilized to examine the morphology and the particle size of the produced N-GQDs. This test provides crucial information on their structural features and potential suitability for crossing the BBB. The prepared N-GQDs possess a quasi-spherical shape and are well-dispersed in an aqueous solution with no agglomeration, as seen in the TEM image in Fig. 1(a and b). The particle size distribution shown in Fig. 1(c) indicates the mean particle size of 7.4 ± 1.7 nm for the N-GQDs.

The small size is a significant factor because fluorescence probes with a small size range (less than 200 nm) offer several benefits for effective transport to the brain for diagnosing AD, including better intracellular permeability, higher solubility in aqueous conditions, enhanced interaction with Aβ plaques, and the capability to penetrate the BBB.^59^ BBB permeability is a crucial necessity for *in vivo* fluorescence imaging; therefore, designing probes with combined nanoscale size and favorable surface chemistry is particularly significant. Compared with metal-based and carbon-based nanoparticles, N-GQDs represent a distinct advantage in achieving precise size control by facile and efficient synthesis routes like pyrolysis. Such size control is challenging for carbon-based nanomaterials such as carbon nanotubes (CNTs) and graphene sheets, as well as for metal nanoparticles, which indicate broader size distributions and tend to aggregate, reducing their efficiency in crossing the BBB.^60^ Among all the nanoparticles, N-GQDs are one of the best candidates for crossing the BBB because of their small size, surface functional groups, and excellent biocompatibility. The TEM results (Fig. 1) verify that their nanoscale size can help with higher BBB penetration.

**Fig. 2:**
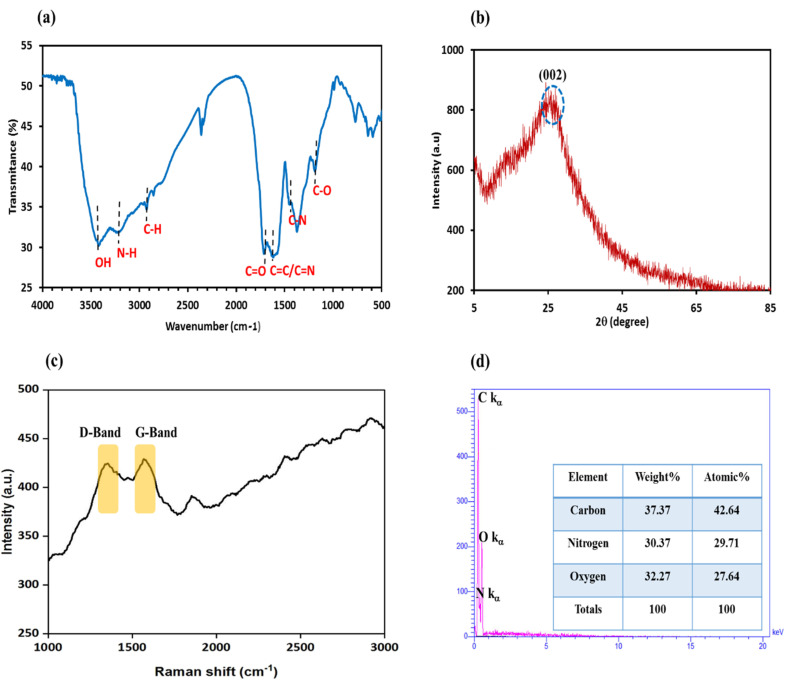
(a) FTIR spectrum of the N-GQDs (b) XRD pattern of the N-GQDs (c) Raman spectrum of the N-GQDs and (d) EDX spectrum of the N-GQDs (inset: quantitative results of presented elements in the N-GQDs).

FTIR spectroscopy was used to verify the presence of surface functional groups on the N-GQDs, which are essential for understanding their surface chemistry and possible interaction mechanism, as seen in Fig. 2(a). Two broad bands observed at 3415 cm^−1^ and 3218 cm^−1^ are due to the O–H and N–H stretching vibrations, confirming the presence of –OH, –COOH, and –NH groups on the N-GQDs.^61,62^ The located peaks at 1716 cm^−1^ and 1623 cm^−1^ are responsible for C=O and C=C/C=N stretching vibrations, respectively.^63,64^ The C–H and C–N stretching vibrations were detected at 2925 cm^−1^ and 1420 cm^−1^, respectively.^65^ Moreover, the absorption peak at 1190 cm^−1^ is related to C–O stretching vibration, confirming the existence of oxygen-rich groups on the obtained N-GQDs surface.^66,67^ The N-GQDs' surface has numerous hydrophilic functional groups, which increase their aqueous dispersibility and stability.^68^ These findings confirm that nitrogen atoms have been effectively incorporated into the structure of GQDs using the current synthesis method.

GQDs indicate superior surface functionalization ability over many metal-based and carbon-based nanomaterials. This is owing to their abundant edge sites, oxygen-containing groups, and a high surface-to-volume ratio, which are readily modified by chemical doping or synthesis approaches. Unlike metal-based nanoparticles such as gold (Au) and silver (Ag), which generally require complex surface modification or ligand-exchange processes to obtain stable functionalization, GQDs naturally provide reactive sites for covalent or non-covalent conjugation.^69^ Additionally, carbon-based nanomaterials like CNTs and graphene sheets commonly have limited dispersibility and fewer reactive functional groups on their basal planes, restricting their functionalization flexibility compared with the edge-rich and defect-abundant structure of GQDs.^70^ These functionalization features directly influence the biological performance of N-GQDs, improving their hydrophilicity and interaction with biological barriers.

This confirmed hydrophilicity is particularly relevant to biomedical purposes. Appropriate lipophilicity is an important feature of BBB permeability, and hydrophilic hydroxyl groups could not only improve lipophilicity value but also enhance brain kinetics. As confirmed by FTIR analysis, the existence of hydroxyl and carboxyl groups in the N-GQDs (Fig. 2(a)) suggests their excellent hydrophilicity. Thus, N-GQDs have good potential for brain uptake, and they are more suitable for brain imaging.

A typical XRD pattern was employed to analyze the crystalline structure of the N-GQDs and to identify the presence of graphitic domains. In Fig. 2(b), the XRD spectrum of the N-GQDs indicates a broad diffraction peak at about 2*θ* = 26.08°, which is attributed to the (002) diffraction planes of the crystal graphitic structure. The nanoscale size of GQDs results in the XRD peak broadening.^71^ Based on Bragg's Law (2*d* sin*θ* = *nλ*, where *n* is diffraction order (*n* = 1), *λ* is the wavelength of the X-ray beam (0.154 nm), *θ* is the position of the (002) peak, and *d* is the interlayer distance), *d*-spacing is approximately 0.34 nm, which is consistent with graphite spacing: 0.335–0.340 nm.^72,73^

Raman spectroscopy was employed to further study the structural features of the N-GQDs, particularly to assess the degree of graphitization and the presence of structural disorders. As shown in Fig. 2(c), the N-GQDs Raman spectrum reveales two characteristic peaks at ∼1370 cm^−1^ (D-band) and ∼1620 cm^−1^ (G-band). The D-band is associated with defects and disorders in amorphous carbon, whereas the G-band is ascribed to the *E*_2g_ vibrational mode of sp^2^ graphitic carbon domains in a two-dimensional hexagonal lattice, indicating crystalline graphitic structures.^74,75^ The peak intensities ratio of D to G bands (*I*_D_/*I*_G_) is an efficient indicator for measuring the graphitization degree of carbon-based materials.^76^ For the prepared N-GQDs, the *I*_D_/*I*_G_ ratio is found to be 0.99, suggesting the presence of a crystalline graphite structure, in agreement with the XRD results.

The EDX characterization was conducted to identify the elemental analysis of the synthesized N-GQDs. This test reveals evidence of nitrogen doping and verifies the presence of expected elements consistent with successful synthesis. The EDX spectrum in Fig. 2(d) proves the presence of carbon, nitrogen, and oxygen elements with quantitative amounts of 37.37%, 30.37%, and 32.27%, respectively; this result indicates that the amount of doped nitrogen is sufficiently high to affect optical properties without causing structural disruption and confirms the N-GQDs' efficient synthesis.

### 3.2. Absorption and fluorescence spectroscopic studies of the N-GQDs

**Fig. 3:**
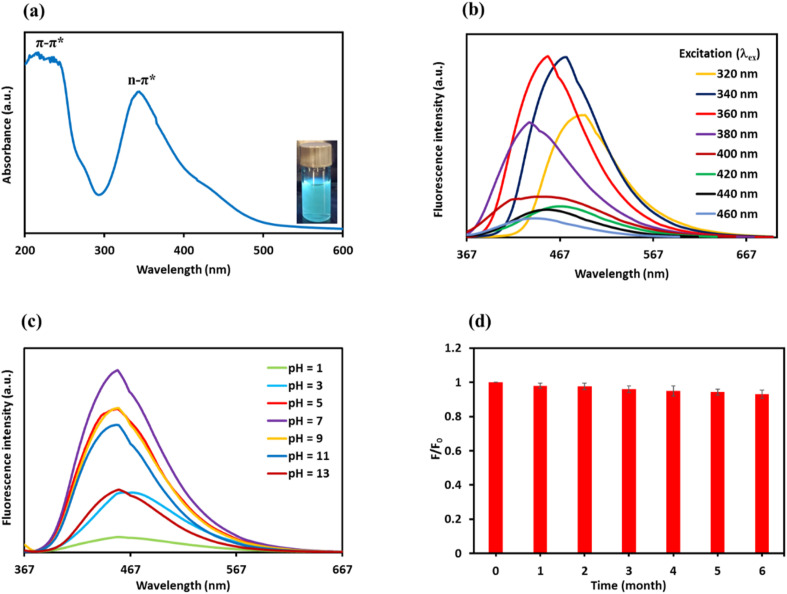
(a) UV-vis absorption spectrum of the N-GQDs (inset: photograph of the N-GQDs solution illuminated by 365 nm UV lamp). (b) The fluorescence emission spectra of the N-GQDs at various excitation wavelengths from 320 to 460 nm. (c) The effect of the pH solution on the fluorescence intensity of the N-GQDs. (d) The influence of storage time on the N-GQDs' fluorescence intensity (*F*/*F*_0_ is the relative fluorescence intensity).

The optical characteristics of the N-GQDs solution were investigated by UV-vis spectroscopy and excitation/emission fluorescence spectra. UV-vis spectroscopy was conducted to assess the optical absorption features of the N-GQDs, which indicate electronic transitions. As illustrated in Fig. 3(a), the UV-vis absorption spectrum exhibits the absorption peak at 218 nm, corresponding to the π–π* transition of aromatic C=C sp^2^ domains, whereas the absorption peak at 344 nm is assigned to the *n*–π* electronic transitions of C=O and C=N bonds.^77^ The insets in Fig. 3(a) also demonstrate that the aqueous N-GQDs solution displays a bright blue color when illuminated by a 365 nm UV lamp.

Fluorescence spectroscopy was performed to investigate the emission behavior of the synthesized N-GQDs and to understand their optical and electronic features. The photoluminescence (PL) spectra were recorded for various excitation wavelengths from 320 nm to 460 nm with 20 nm intervals (Fig. 3(b)). The fluorescence spectra of the N-GQDs in Fig. 3(b) indicate that as the excitation wavelength shifted from 320 to 360 nm, the fluorescence emission intensity increased and then decreased for excitation wavelengths 380 to 460 nm. The highest emission intensity was recorded at a wavelength of 454 nm under an excitation wavelength of 360 nm. This result revealed that the N-GQDs had an excitation-dependent fluorescence emission, which arises from the different particle size distribution and defect states. The size variation of the N-GQDs creates discrete sp^2^-related localized states at the LUMO and HOMO levels. The electronic transitions from these localized states lead to shifts in the position of the PL emission peak. In addition, the presence of various edge functional groups in the N-GQDs, such as oxygen and nitrogen atoms, can introduce trap states between the LUMO and HOMO energy levels, resulting in emission that depends on the excitation wavelength.^78^ The QY of prepared N-GQDs was measured to be 57.3%.

GQDs exhibit several advantages in fluorescence properties over both metal- and other carbon-based nanomaterials. Their fluorescence is highly tunable due to quantum confinement and edge effects, enabling controlled emission across a wide spectral range. In comparison to carbon quantum dots (CQDs), GQDs typically show higher QY and PL efficiency because of their well-ordered crystalline structure, contributing to better fluorescence performance. Unlike metal-based quantum dots, which often suffer from fluorescence quenching, GQDs provide stable fluorescence emission and resistance to photobleaching. Moreover, N-doping further improves the electronic structure and QY, thereby producing more stable fluorescence emission.^79^ These features make N-GQDs particularly suitable for fluorescence-based sensing, including sensitive detection of Aβ aggregates in AD studies.

The fluorescence intensity of the N-GQDs was examined in a wide pH range from 1 to 13 to evaluate their optical response in varying pH conditions. The N-GQDs emission intensity progressively increased from strong acidic conditions (pH 1) up to neutral pH (pH 7), and it reached its maximum value at neutral pH, as seen in Fig. 3(c). On the other hand, in alkaline conditions (pH 9–13), a significant decrease in emission intensity was observed with increasing pH, especially in strong basic conditions. These results demonstrated that a strongly acidic and alkaline condition unfavorably affects the emission intensity of the N-GQDs. The decrease of fluorescence intensity in strongly acidic and alkaline conditions can be attributed to alterations in surface charge generated by the protonation and deprotonation of the functional groups on the N-GQD surface.^80^ Therefore, pH 7 with the optimum fluorescence signal was selected for the subsequent studies.

Photostability analysis was carried out to examine the long-term stability of the fluorescence emission of the N-GQDs solution used in the experiments. The fluorescence intensity at 454 nm wavelength was recorded for up to 6 months. The N-GQDs solution was placed in the refrigerator at 4 °C following each experiment. The results in Fig. 3(d) exhibit that the N-GQDs emission intensity remains without apparent change after 6 months. The deviations of fluorescence intensity are less than 8%, confirming that the N-GQDs possessed superb photostability. It is important to note that the N-GQDs maintained stability in aqueous solution without signs of aggregation or loss of their fluorescence even after being stored for several months.

This outstanding photostability distinguishes N-GQDs from many metal-based and other carbon-based nanomaterials. Metal nanoparticles such as indium phosphide (InP)/zinc selenide (ZnSe)/zinc sulfide (ZnS) quantum dots often undergo photobleaching or surface oxidation under UV illumination,^81^ reducing emission efficiency, while the metal-free N-GQDs exhibit much higher photostability due to their distinct electronic structure. Moreover, carbon-based nanomaterials like graphene oxide (GO) and fullerene demonstrate weak photostability compared to N-GQDs.^82,83^ Importantly, most probes currently used for *in vivo* imaging of Aβ plaques are fluorescence dyes, which suffer from limited photostability owing to photobleaching. Consequently, N-GQDs with excellent photostability can be considered diagnostic agents in long-term bioimaging studies.

### 3.3. Cytotoxicity investigation of the N-GQDs

**Fig. 4:**
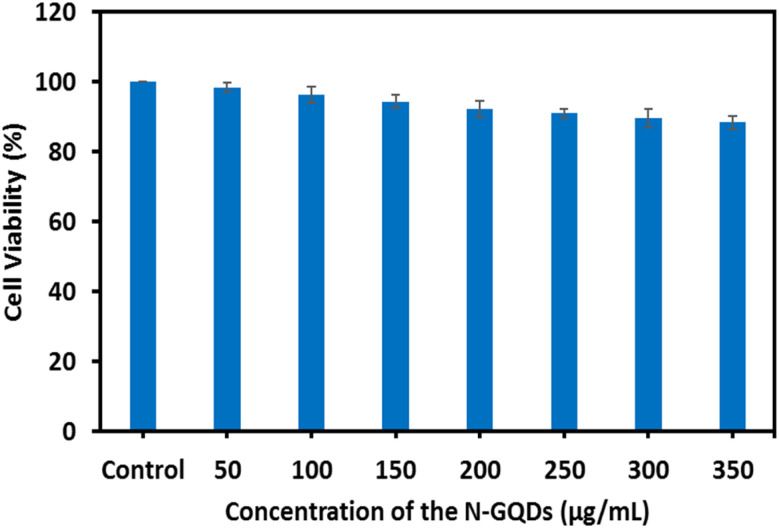
The viability of PC12 cells with various concentrations of the N-GQDs (50–350 µg mL^−1^) after incubation for 24 h (the data are shown with the mean ± standard deviation).

The toxicity of nanomaterials plays a vital role in their biological purposes. Thus, to investigate the toxicity of the N-GQDs on PC12 cell lines, the MTT viability assay was conducted. Cells were incubated with various concentrations of N-GQDs from 50 to 350 µg mL^−1^ for 24 h. As shown in Fig. 4, even at the highest concentration (350 µg mL^−1^), N-GQDs did not cause significant cytotoxic effects, and cell viability remained above 85% compared with the untreated control. According to these findings, the N-GQDs possess low cytotoxicity and excellent biocompatibility.

The cytotoxicity evaluation of fluorescence probes is essential for understanding their potential applications in biomedical fields. Compared to conventional metal-based nanoparticles such as Au, Ag, or semiconductor quantum dots like cadmium selenide zinc sulfide (CdSe/ZnS), N-GQDs indicate superior biocompatibility and lower cytotoxicity. This advantage arises because N-GQDs lack heavy metals that can trigger oxidative stress, DNA damage, and organ toxicity, making them safer alternatives for *in vivo* usage.^84^ A few previous studies have discussed the *in vivo* toxicity of probes. For instances, Nurunnabi *et al.* investigated the *in vivo* toxicity of GQDs, and their results showed that GQDs were removed from the body after 24 h, confirming the low *in vivo* cytotoxicity of the GQDs.^35^ Consistent with these findings, our *in vitro* results further support the safety of N-GQDs, which is owing to their small size and oxygen content. Together, these features indicate their potential as an excellent fluorescent probe for bioimaging studies and promise for the clinical diagnosis of AD.

### 3.4. Fluorescence response of the N-GQDs toward Aβ_25–35_ aggregates

**Fig. 5:**
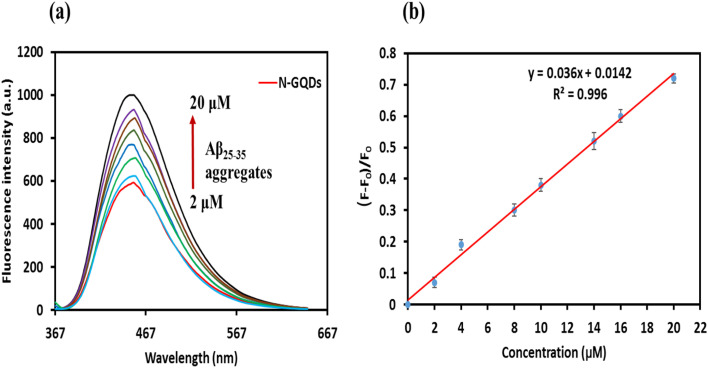
(a) Fluorescence behavior of N-GQDs (0.1 mg mL^−1^) with various concentrations of Aβ_25–35_ aggregates. (b) The linear correlation between the [(*F*–*F*_0_)/*F*_0_] and the concentration of Aβ_25–35_ aggregates within the range of 2–20 µM (*F*_0_ and *F* show the fluorescence intensities of the N-GQDs without and with Aβ_25–35_ aggregates, respectively). The data are indicated with the mean ± standard deviation (*n* = 3).

A fluorescent probe that effectively binds Aβ should display noticeable changes in its fluorescence features upon interaction with Aβ aggregates. Thus, we evaluated the fluorescence behavior of the N-GQDs with various concentrations of Aβ_25–35_ aggregates. Fig. 5(a) shows the fluorescence enhancement ratio [(*F*–*F*_0_)/*F*_0_] with the concentration of Aβ_25–35_ aggregates, in which *F*_0_ and *F* represent the fluorescence intensities of the N-GQDs without and with Aβ_25–35_ aggregates, respectively. The fluorescence intensities indicated a linear dependence on the concentration of Aβ_25–35_ aggregates in the 2–20 µM range, as seen in Fig. 5(b). The linear regression is described by the equation *y* = 0.036*X* + 0.0142, where *X* is the concentration of Aβ_25–35_ aggregates, and the corresponding regression coefficient was 0.996. Furthermore, this technique demonstrated a detection limit of 1.6 µM for Aβ_25–35_ aggregates.

The N-GQDs fluorescence emission progressively increased as the concentration of Aβ_25–35_ aggregates was raised (Fig. 5(a)). The fluorescence enhancement may result from the motion restriction of the photo-excited N-GQDs when bound to Aβ because Aβ_25–35_ aggregates consume the N-GQDs' active interaction sites and contact surface areas, leading to a non-radiative decay rate decrease. These results suggested that our probe has a high sensitivity to Aβ_25–35_ aggregates and can be selectively “turned on” upon binding with it. This interaction supported by surface characteristics of the N-GQDs, which are crucial in their chemical interaction with Aβ plaques. Small nanoparticles, like our probe, with high surface areas have a stronger affinity for Aβ aggregates. The surface of N-GQDs possesses oxygen- and nitrogen-based functional groups, which facilitate their interactions with Aβ aggregates *via* mechanisms like hydrophobic interaction, hydrogen bonding, and electrostatic interaction. The oxygen-containing groups on the surface of N-GQDs provide hydrogen bonding and electrostatic interactions, further stabilizing the complex with Aβ_25–35_. Moreover, N-doping introduces localized negative charges and electron-rich sites on graphene quantum dots, boosting their affinity for positively charged amino acids in Aβ_25–35_.

The observed fluorescence increase *in vitro* (Fig. 5(a)) can be explained by the interaction of hydrophobic residues in Aβ aggregates with the hydrophobic basal plane in N-GQDs. This interaction restricts the rotation of N-GQDs after excitation and decreases the non-radiative decay rate, consequently enhancing fluorescence intensity. Our fluorescence data verified that N-GQDs have high selectivity and sensitivity to Aβ_25–35_ aggregates. Previous studies, such as Huang *et al.*, reported that GQDs could detect monomeric Aβ by fluorescence spectroscopy. They showed the linear relationship between monomeric Aβ concentration and the fluorescence intensity of GQDs.^85^ There is no evidence regarding the *in vivo* detection of monomeric Aβ with GQDs in physiological environments. Therefore, the ability of the N-GQDs to detect Aβ_25–35_ aggregates can provide excellent opportunities for the design of novel fluorescent probes for Aβ plaque imaging both *in vivo* and *ex vivo*.

### 3.5. Selectivity of N-GQDs toward different Aβ species

**Fig. 6:**
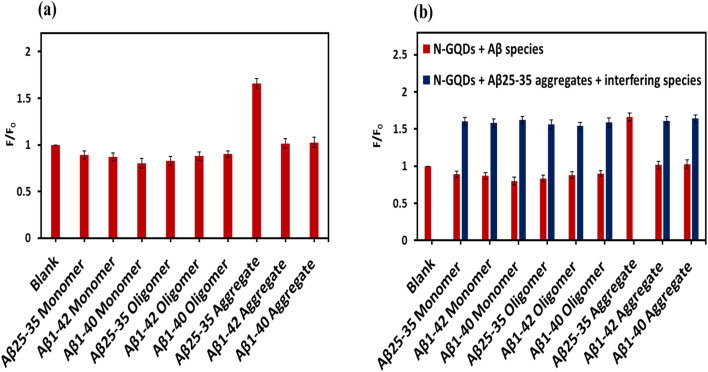
(a) Selectivity of the N-GQDs (0.1 mg mL^−1^) for Aβ_25–35_ aggregates (10 µM) over the other Aβ species (10 µM each). (b) Fluorescence response of N-GQDs with Aβ_25–35_ aggregates (10 µM) in the presence of various interference Aβ species, each at 10 µM (blue bars) (*F*_0_ and *F* indicate the fluorescence intensities of the N-GQDs without and with Aβ species, respectively; Blank: N-GQDs only). The results are demonstrated with the mean ± standard deviation (*n* = 3).

To investigate the selectivity of N-GQDs toward Aβ_25–35_ aggregates, fluorescence experiments were performed with possible interference Aβ species, including Aβ_1–42_ and Aβ_1–40_ in their monomeric, oligomeric, and aggregated forms. Fig. 6 demonstrates the relative change of the fluorescence intensity (*F*/*F*_0_) of N-GQD with various Aβ species, each at an identical concentration of 10 µM. According to Fig. 6(a), N-GQDs demonstrated a significant fluorescence enhancement selectively in the presence of Aβ_25–35_ aggregates, with *F*/*F*_0_ value of 1.65 relative to the blank (*F*/*F*_0_ = 1). In contrast, the addition of monomeric or oligomeric forms of Aβ_25–35_ resulted in only negligible changes in fluorescence intensity, yielding *F*/*F*_0_ values of 0.89 and 0.83, respectively. These results indicate that the fluorescence response of N-GQDs is highly conformation-dependent.

Similarly, when N-GQDs were incubated with monomers, oligomers, and aggregates of Aβ_1–40_ and Aβ_1–42_, no obvious fluorescence variations (*F*/*F*_0_ ranged from 0.8 to 1.03) were detected compared with the pronounced fluorescence enhancement observed for Aβ_25–35_ aggregates (Fig. 6(a)). These results clearly suggest that the fluorescence behavior of N-GQDs is selective toward the aggregated form of Aβ_25–35_.

Moreover, to further confirm selectivity under competitive conditions, fluorescence measurements were also conducted in the presence of Aβ_25–35_ aggregates together with other Aβ species. The coexistence of Aβ_1–40_ or Aβ_1–42_ (in monomeric, oligomeric, or aggregated forms) caused only a minor change in the fluorescence enhancement produced by Aβ_25–35_ aggregates (Fig. 6(b), blue bars). This observation suggests that other Aβ species do not markedly interfere with the interaction between N-GQDs and Aβ_25–35_ aggregates.

The observed selectivity can be attributed to structural and physicochemical differences among the Aβ species. Aβ_25–35_ is known to form compact, highly hydrophobic aggregates with exposed β-sheet domains, which may promote strong π-stacking, hydrogen bonding, and hydrophobic interactions with the surface functional groups and the sp^2^ carbon domains of N-GQDs. In contrast, Aβ_1–40_ and Aβ_1–42_ possess long peptide sequences with various aggregation morphologies and surface charge distributions, resulting in weaker or negligible interactions with the N-GQDs surface. Consequently, these findings confirm that N-GQDs exhibit good selectivity toward Aβ_25–35_ aggregates over other Aβ species, highlighting their potential application as selective fluorescent probes for the detection of toxic Aβ_25–35_ aggregates.

### 3.6. Molecular docking analysis of N-GQDs and Aβ_25–35_ aggregates

**Fig. 7:**
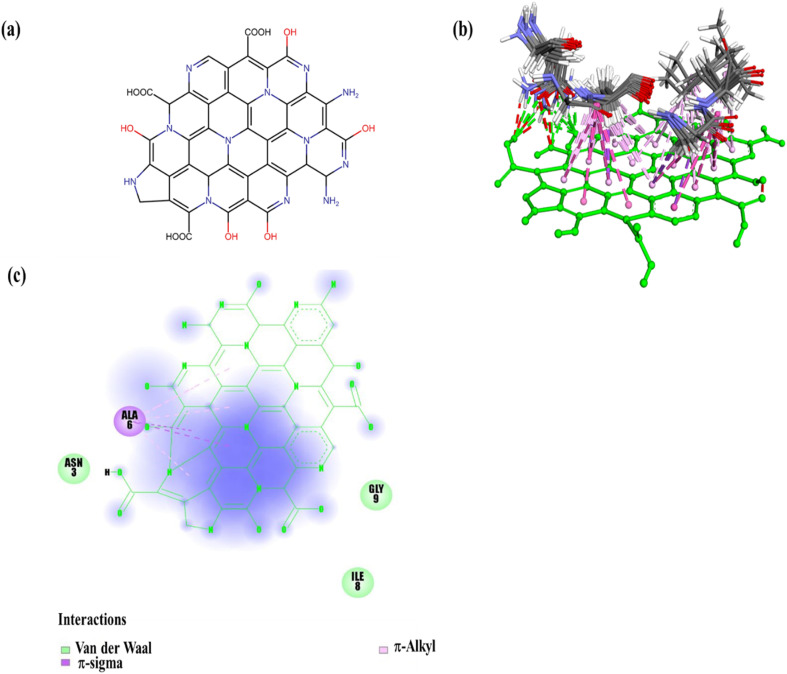
(a) Proposed chemical structure of the N-GQDs. (b) Predicted binding interactions between the N-GQDs and Aβ_25–35_ aggregates. (c) π-sigma and π-alkyl interactions of the ALA6 residue of the Aβ_25–35_ aggregates with the surface of the N-GQDs.

The binding mechanism of the N-GQDs with Aβ_25–35_ aggregates was investigated through molecular docking. Discovery Studio and ChimeraX were employed to analyze the binding energy and other parameters, such as hydrogen bonds, electrostatic interactions, and van der Waals interactions. Molecular docking analysis indicated several key interactions between N-GQDs and Aβ_25–35_ aggregates, and Table 1 lists them. Fig. 7 shows the proposed structure of N-GQDs and their contact model with Aβ_25–35_ aggregates. According to docking analysis, hydrogen bonds were created between the surface functional groups of the N-GQDs and key residues of Aβ_25–35_, including ASN3:HD22 and ASN3:OD1. These hydrogen bonds may improve the selective binding affinity and support the stability of the N-GQDs-Aβ_25–35_ complex. Detailed investigation of the binding mode indicated that the hydrophilic domain on the N-GQDs is a significant factor in facilitating interaction with Aβ_25–35_, thereby increasing binding affinity *via* hydrogen bonding.

**Table 1:** Types of molecular interactions observed in docking of the N-GQDs with Aβ_25–35_ aggregates

Interactions	Distance (A°)	Category	Type
ASN3:HD22 – N-GQDs:O4	1.84	Hydrogen bond	Conventional hydrogen bond
ASN3:OD1 – N-GQDs:H10	2.62	Hydrogen bond	Conventional hydrogen bond
ALA6:HA – N-GQDs	2.92	Hydrophobic	π-sigma
GLY9:HA2 – N-GQDs	2.92	Hydrophobic	π-sigma
LEU10:HD22 – N-GQDs	2.14	Hydrophobic	π-sigma
GLY5:C,O; ALA6:N – N-GQDs	5.08	Hydrophobic	Amid-π stacked
GLY9:C,O; LEU10:N – N-GQDs	4.20	Hydrophobic	Amid-π stacked
LEU10 – N-GQDs	5.15	Hydrophobic	π-Alkyl
ALA6 – N-GQDs	5.35	Hydrophobic	π-Alkyl
LEU10 – N-GQDs	5.25	Hydrophobic	π-Alkyl
LEU10 – N-GQDs	4.86	Hydrophobic	π-Alkyl

In addition to polar interactions, we observed several hydrophobic interactions, such as amide-π stacking, π-sigma, and π-alkyl interactions, between N-GQDs and the nonpolar amino acid residues of Aβ_25–35_ aggregates. These further improve the overall stability and specificity of the complex. Docking results demonstrated that the N-GQDs formed amide-π stacked interactions with the backbone amide groups of Gly5:C, O, and AlA6:N, and π-alkyl interactions with side chains of Gly9:C, O, and LEU10:N, stabilizing the complex. Additionally, π-sigma interactions were observed between the aromatic rings of the N-GQDs and the ALA6:HA, GLY9:HA2, and LEU10:HD22 of Aβ_25–35_. As demonstrated in Fig. 7(c), the ALA6 residue of the Aβ_25–35_ aggregates forms π-sigma and π-alkyl interactions with the surface of the N-GQDs. These interactions are critical for specific binding to Aβ, suggesting stable and specific binding relevant for diagnostic applications.

The best docking pose (conformer 1) displayed a binding score of −57.4 kcal mol^−1^, indicating a strong and stable predicted binding affinity. This finding is more favorable than values reported in previous studies. For comparison, Wu *et al.*^86^ reported a binding energy of −6.753 kcal mol^−1^ for CAQ interacting with Aβ_1–42_ aggregates, while Rajasekhar *et al.*^87^ observed a binding energy of −9.86 kcal mol^−1^ for the interaction of hemicyanine-based benzothiazole-coumarin (TC) with Aβ_42_ aggregates. Although the differences in docking methodologies across studies may affect the absolute values of binding energies, our fluorescence experimental data confirm the significance of these findings. This remarkably high affinity can be attributed to nitrogen-induced modulation of the GQD electronic structure, which increases the density of active sites and enhances hydrogen bonding and π-interactions with amyloid aggregates. To our knowledge, this is the first report demonstrating such a high binding energy between N-GQDs and Aβ aggregates, highlighting nitrogen doping as a key factor in improving molecular recognition and binding stability. This finding of molecular docking suggests why N-GQDs are an Aβ_25–35_ detector and provides crucial guidelines for designing Aβ sensing probes.

### 3.7. Neurobehavioral evaluation (MWM)

**Fig. 8:**
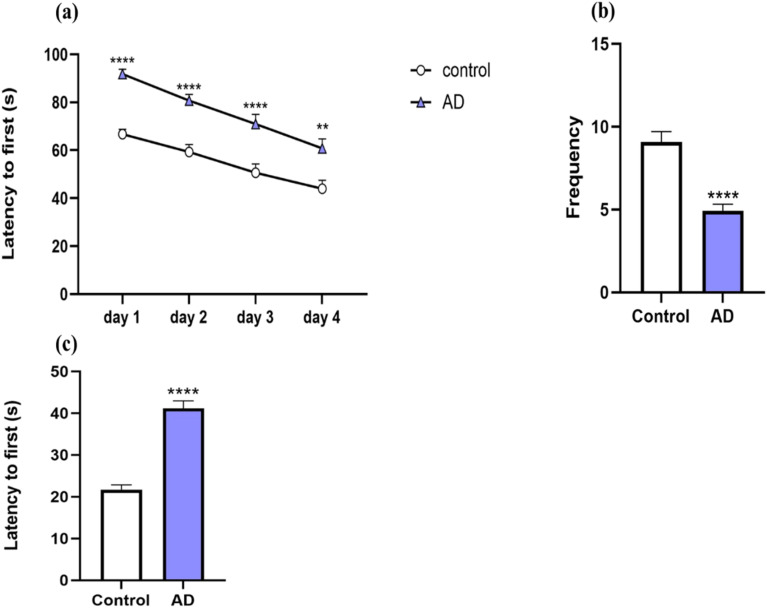
The effect of ICV injection of Aβ_25–35_ aggregates on learning and memory performance in rats in the MWM test. (a) Latency to first reaching the hidden platform during four consecutive trial days in the AD model group and the control group (b) the number of crossings of the platform during the probe trial test in the AD model group and the control group (c) latency to first during the probe trial test in the AD model group and the control group. To assess the significant difference between groups, *p < 0.05, **p < 0.01, ***p < 0.001, and ****p < 0.0001 were compared with the control group. The data are displayed as mean ± standard error of the mean (SEM) (*n* = 12 rats in each group).

The AD model was created by ICV administration of Aβ_25–35_ aggregates. To investigate the performance of spatial learning and memory in the study groups, we employed the MWM test. Fig. 8(a) shows the latency to reach the hidden platform in the MWM acquisition phase for both the AD model group and the control group. Rats in the AD model group needed significantly more time to locate the platform than those in the control group, as illustrated in Fig. 8(a). Moreover, on day four, latency in the AD model group was noticeably greater than in the control group (*p* < 0.01). The results of the probe trial test for the frequency and latency to first are shown in Fig. 8(b and c). The AD model group exhibited a notable decrease in the number of platform crossings compared with the control group (*p* < 0.0001) (Fig. 8(b)). As seen in Fig. 8(c), the latency in the probe trial for the AD model group was markedly increased relative to the control group (*p* < 0.0001). These results indicate that Aβ_25–35_ aggregates attenuate memory and learning in normal rats.

### 3.8. Congo red staining for the demonstration of the Aβ plaque deposits

**Fig. 9:**
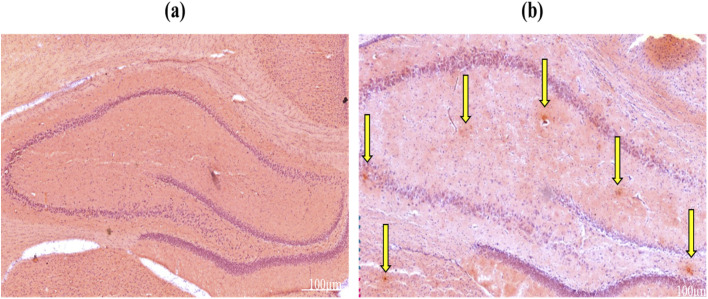
Congo red staining method for Aβ plaque accumulation in the hippocampus region for (a) the control group and (b) the Aβ_25–35_ injected group (*n* = 4 per group). Yellow arrows display Aβ plaques.

The accumulation of Aβ aggregates within the hippocampus and neocortex is a significant factor in the progression of AD pathology.^88^ Congo red staining was employed to verify the existence of Aβ plaques in brain sections of the AD model. Fig. 9 indicates Aβ plaque formation in the hippocampus. As seen in Fig. 9(a), the control group had no Aβ deposits in the hippocampus. The findings showed that Aβ_25–35_ ICV injection in healthy rats led to significant Aβ peptide deposition (Fig. 9(b)).

### 3.9. *In vivo* imaging of Aβ plaques with N-GQDs in the AD rat model

**Fig. 10:**
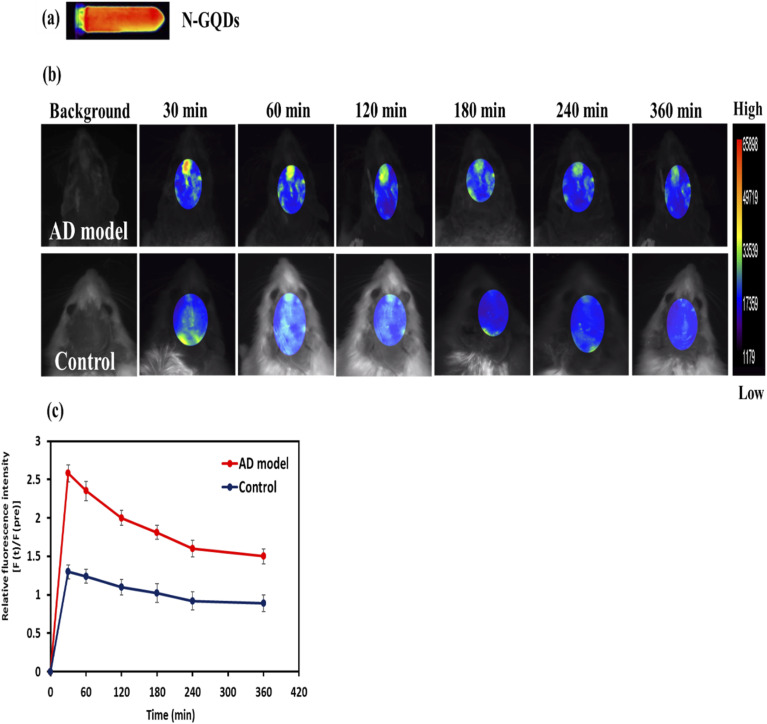
(a) Optical image of the N-GQDs. (b) *In vivo* fluorescence images of AD model rat and control rat at various time points before and after intravenous injection of the N-GQDs (5 mg kg^−1^, pH = 7.4) (excitation/emission wavelength = 470/535 nm). (c) The plot of the relative fluorescence intensity [*F*(*t*)/*F*(pre)] of AD model rats and control rats (*n* = 4 per group) after intravenous injection of the N-GQDs at various time points. The relative fluorescence intensities [*F*(*t*)/*F*(pre)] of AD model rats were notably stronger than in the control rats (***p < 0.001). The findings are shown with the mean ± standard deviation.

The fluorescent probe used for *in vivo* optical imaging should meet some requirements, including reasonable washout kinetics, low physiological toxicity, and logical BBB penetration. Moreover, the rapid clearance of the unattached probe in the target tissues will increase the signal-to-noise ratio.^89^ To assess whether N-GQDs could detect Aβ plaques *in vivo* using fluorescence imaging, we employed the AD model rats and age-matched control rats. The fluorescence signals from the brains of the AD model rats were notably greater than in the age-matched control rats at every time point after intravenous injection of the N-GQDs, as seen in Fig. 10(b). Time-dependent decreases in fluorescence signals in Fig. 10(b) indicate that the small-sized N-GQDs might be gradually eliminated from the brain, revealing that N-GQDs have highly desirable features for fluorescence imaging because of their logical washout. To carry out a semi-quantitative evaluation, fluorescence intensity was normalized at each time [*F*(*t*)] against background fluorescence intensity before injection [*F*(pre)] (Fig. 10(c)). The signals of the AD model rats were 1.98-, 1.89-, 1.81-, 1.77-, 1.73-, and 1.68-fold higher than the signal of age-matched control rats at 30, 60, 120, 180, 240 and 360 min, respectively (Fig. 10(c)). The stronger fluorescence signals in the brains of the AD model rats may be due to the binding of the N-GQDs with Aβ plaques. These results imply that N-GQDs were capable of detecting Aβ plaques *in vivo*.

The detection of Aβ plaques *in vivo* has significant implications for AD diagnosis, monitoring, and assessing the effectiveness of treatments. A non-invasive imaging technique makes it possible to visualize and measure Aβ accumulations in patients, offering a crucial biomarker that complements clinical and cognitive evaluations. Early and precise detection of these plaques facilitates differential diagnosis and can identify individuals in preclinical or mild cognitive impairment stages, allowing for timely intervention. This early detection is essential because pathological changes happens decades before symptoms appear. Furthermore, Aβ plaque detection over time is an effective tool for disease monitoring, as increasing plaque density correlates with advancing stages of AD. This progression tracking is crucial for understanding disease dynamics and cognitive decline. The ability to monitor these plaques *in vivo* is invaluable for both clinical management and clinical trials.^90,91^

From a therapeutic perspective, Aβ detection is essential for evaluating the efficacy of emerging anti-Aβ therapies. Many investigational treatments aim to decrease amyloid accumulation or facilitate its clearance. For instance, Aducanumab, as an anti-Aβ drug, has demonstrated the potential to decrease Aβ plaques in early AD or mild cognitive impairment. Therefore, *in vivo* imaging methods that measure Aβ plaque before and after treatment administration provide the investigation of therapy response and optimization of clinical protocols.^92^

Conventional neuroimaging methods for the prediction and diagnosis of AD, including PET, SPECT, and MRI, have been used; nevertheless, non-invasive fluorescence techniques for the *in vivo* detection of AD biomarkers can make it easier to investigate the disease prediction and/or progression in AD animal models and may lead to an earlier diagnosis in the human who is prone to AD. Regarding this, the use of various probes for fluorescence imaging of Aβ species, such as AOI-987,^23^ CRANAD-2,^24^ THK-265,^93^ CRANAD-58,^25^ CRANAD-3,^26^ and PTO-29,^94^ has been reported. The majority of these probes cannot meet requirements for imaging Aβ species, such as high fluorescence intensity upon binding with Aβ species, low cytotoxicity, high selectivity towards Aβ species, reasonable lipophilicity, as well as good BBB permeability. Of these probes, the high lipophilicity of CRANAD-2 and CRANAD-58 causes low BBB penetration, which makes it harder to diagnose AD clinically. Therefore, research into discovering better and more functional probes is still ongoing. In response to this necessity, our group decided to develop a novel fluorescence probe based on N-GQDs for *in vivo* imaging of Aβ aggregates to solve diagnostic challenges related to AD. Based on our primary knowledge, the N-GQDs appear to be the best candidate for achieving this purpose. Recent studies on GQD-based probes for AD have reported on therapeutic applications. In contrast, our study addresses a crucial gap by developing a GQD probe optimized specially for sensitive and selective diagnosis of Aβ aggregates *in vivo*. To emphasize the differences, we compared our N-GQDs with reported GQDs-based probes for AD in the current research (Table 2). Table 2 summarizes representative studies from the literature that compare probe types, targets, experimental models, and their main applications, whether diagnosis or therapeutic.

**Table 2:** Comparison of reported GQDs-based probes for AD-related applications

Probe type	Target	Model (*in vitro*/*in vivo*)	Detection limit	Mechanism validation	Application (diagnosis/therapeutic)	References
GQDs	—	*In vivo*	—	—	Therapeutic	95
GQDs	Aβ_42_ monomers, oligomers, and fibrils	*In vitro in silico*	—	Molecular dynamics simulations	Therapeutic	96
N,F-GQDs	Aβ_42_ fibrils	*In vitro*	—	—	Therapeutic	44
GQDs	Tau aggregates	*In silico*	—	Molecular docking	Therapeutic	97
Chitosan/GQDs	—	*In vivo*	—	—	Therapeutic	98
N-GQDs-Memantine	Aβ_1–42_ aggregates	*In vitro*	—	—	Therapeutic	36
GQDs	Aβ_42_ aggregates	*In vitro*/*in silico*	—	Molecular dynamics	Therapeutic	45
GQDs@MPN	Aβ_42_ aggregates	*In vivo*	—	—	Therapeutic	99
N-GQDs	Aβ_25–35_ aggregates	*In vitro*/*in vivo*/*ex vivo*/*in silico*	1.6 µM	Molecular docking	Diagnosis	This work

As shown in Table 2, current studies have focused on therapeutic modalities, while our N-GQDs are designed as diagnostic imaging probes for Aβ *in vivo*. Therefore, quantitative therapeutic metrics (such as cognitive function improvement or plaque size decrease) from therapeutic studies are not directly comparable with diagnostic sensitivity measurements, like fluorescence enhancement and detection limits. This comparative analysis further indicates the critical need for highly sensitive and selective diagnostic agents that can detect early AD, which is addressed in our study.

To validate the ability of our probe for *in vivo* detection of Aβ aggregates, we performed experiments with the AD model rat. In this work, we established the AD model by ICV injection of Aβ_25–35_ aggregates. The AD model was confirmed by behavioral assessment (Fig. 8) and histological confirmation (Fig. 9). For *in vivo* fluorescence imaging, we examined N-GQDs with several wavelengths. Unlike previous research that utilized longer wavelengths for *in vivo* imaging, we optimized our imaging protocol using excitation/emission wavelengths at 470/535 nm, where the N-GQDs exhibit the best fluorescence efficiency. We observed that the fluorescence signals in AD model rats were higher compared with control rats (Fig. 10(b)). This enhancement is likely due to the specific interaction of the N-GQDs with Aβ plaques in the AD model rats *in vivo*. These *in vivo* findings are consistent with our *in vitro* binding investigation findings, which showed high selectivity of N-GQDs for Aβ_25–35_ aggregates. In contrast to CRANAD-based probes, which suffer from high lipophilicity and limited BBB penetration, N-GQDs demonstrate efficient brain penetration, confirmed by *in vivo* studies. To our knowledge, this research is the first to offer comprehensive evidence that N-GQDs can detect Aβ plaques non-invasively in both *in vitro* and *in vivo*, marking a clear distinction from previous GQD-based therapeutic studies. Our results suggest that fluorescence imaging with N-GQDs can provide a new approach for early and accurate diagnosis of AD pathology.

### 3.10. *Ex vivo* biodistribution imaging of Aβ plaques with N-GQDs in the AD rat model

**Fig. 11:**
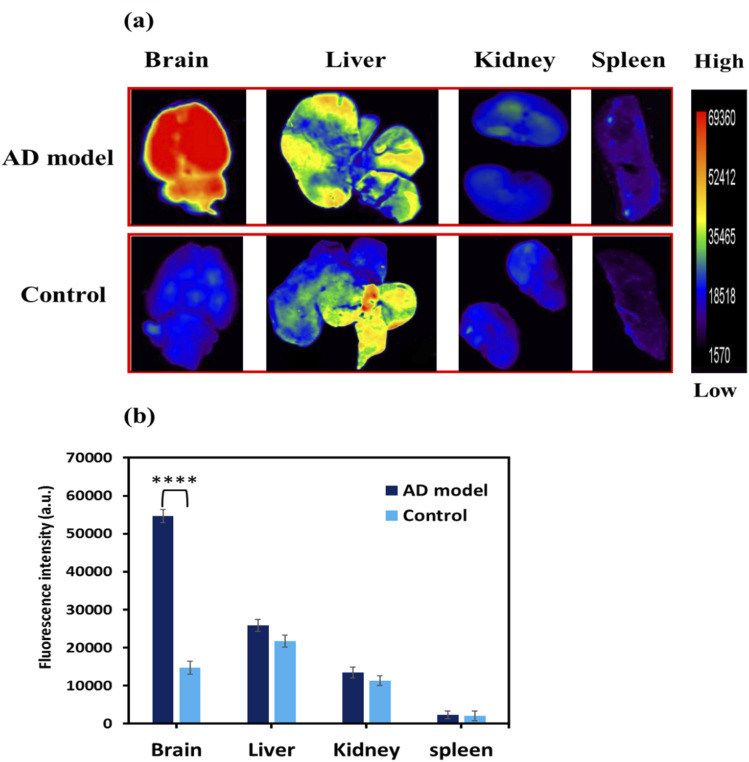
(a) Fluorescence images of the extracted brain and internal organs (liver, kidney, and spleen) of AD model rat and control rat after 30 min intravenous injection of the N-GQDs (5 mg kg^−1^, pH = 7.4). (b) The plot of fluorescence intensity of *ex vivo* brain and internal organs of AD model rats and control rats (*n* = 4 per group). The fluorescence intensity of the brain in the AD model rats was greater than in the control rats (****p < 0.0001).

The *ex vivo* images of different isolated organs were also examined by fluorescence imaging to assess the biodistribution of intravenously injected N-GQDs. To measure the fluorescence intensities in the main internal organs, the AD model rats and age-matched control rats were sacrificed 30 min after the intravenous injection. As seen in Fig. 11(a), the *ex vivo* brain of the AD rat model demonstrated a notable increase in fluorescence intensity compared with the age-matched control rat. This *ex vivo* biodistribution comparative assay revealed that N-GQDs accumulate specifically in the AD model rat brain and non-specifically in the control rat brain (Fig. 11(a)). Fig. 11(b) indicates that a smaller amount of the probe was distributed in other organs, such as the kidneys and spleen. Moreover, according to Fig. 11(a) and (b), N-GQDs are metabolized in the liver and are excreted by the kidneys. This finding suggests favorable clearance pathways and confirms that they do not show significant neurotoxicity at high doses. The *ex vivo* biodistribution showed that N-GQDs could bind to Aβ plaques, which indicated that this result agrees with the *in vivo* imaging data.

Although the present study demonstrates the potential of N-GQDs for highly selective and non-invasive detection of Aβ aggregates, several aspects merit further investigation. The long-term biocompatibility, pharmacokinetics, and potential immune responses of N-GQDs *in vivo* require evaluation. Furthermore, extending the application of this platform to human samples or advanced transgenic AD models could further verify its clinical applicability. Future studies may explore targeted surface functionalization or heteroatom co-doping approaches to improve diagnostic accuracy and multimodal imaging ability. These efforts would facilitate the clinical potential of N-GQDs as next-generation diagnostic probes for AD.

## 4 Conclusion

In conclusion, we successfully designed a new fluorescence probe using N-GQDs for the detection of Aβ plaques in the AD model rat. N-GQDs were produced by a facile carbonization and pyrolysis approach, indicating a nanoscale size of approximately 7.4 nm and a high QY of 57.3%. They demonstrated excellent biocompatibility with cell viability exceeding 90% even at 250 µg mL^−1^, confirming their low cytotoxicity. The probe exhibited high selectivity and increased fluorescence signal upon binding with Aβ_25–35_ aggregates, allowing a detection limit as low as 1.6 µM in *in vitro* studies. In addition, molecular docking analysis confirmed the strong binding interaction energy between N-GQDs and Aβ_25–35_ aggregates (−57.4 kcal mol^−1^) at the molecular level, offering insight into the N-GQDs' mechanism of action. More importantly, the N-GQDs showed excellent *in vivo* imaging features, including strong fluorescence emission, effective BBB penetration, low cytotoxicity, and high biocompatibility. Employing the fluorescence imaging technique, Aβ plaques in the AD model rats were detected after the intravenous injection of N-GQDs, and the fluorescence signal in the brain of the AD model rats was nearly 2-fold stronger relative to the control group. N-GQDs demonstrated the specific interaction with Aβ plaques in the AD model rat *in vivo*. These observations were further verified by the *ex vivo* fluorescence imaging of the extracted brain and other organs. These findings suggest the potential of N-GQDs as a fluorescence imaging probe for early detection and real-time monitoring of AD progression.

## Ethical statement

The present research is ethically approved by the Ethics Committee of the Iran University of Medical Sciences (ethical code: IR.IUMS.AEC.1403.045).

## Author contributions

Mina Bahman: writing – original draft, software, investigation, formal analysis, data curation. Mohammad-Reza Milani-Hosseini: supervision, methodology, validation, visualization. Seyed Behnamedin Jameie: writing – review & editing, conceptualization, project administration, validation, resources.

## Conflicts of interest

The authors declare no conflicts of interest.

## Data Availability

The authors verify that the data supporting the findings of this study are available within the article. The data are also accessible from the corresponding author upon reasonable request.
